# Supplementary material for: Unveiling the Bioactive Potential of the Invasive Jellyfish Phyllorhiza punctata Through Integrative Transcriptomic and Proteomic Analyses
Source: Biomolecules. 2025 Aug 4;15(8):1121. doi: 10.3390/biom15081121 (PMC12383608; doi:10.3390/biom15081121)
Supplement: Supplementary file 1 [file biomolecules-15-01121-s001.zip › Suplementary Material/FileS3/PEPFOLD4/XP_065052404_2046/clusters.html]

 PEP-FOLD clusters


Download archive of all models  here   

|  |  |  |
| --- | --- | --- |
| # | file | sOPEP |
| # cluster 1 - Size: 7 |  |  |
|  | XP\_065052404\_2046-model1 | -20.7393 |
|  | XP\_065052404\_2046-model1.1 | -19.9945 |
|  | XP\_065052404\_2046-model1.2 | -19.662 |
|  | XP\_065052404\_2046-model1.3 | -18.7567 |
|  | XP\_065052404\_2046-model1.4 | -18.3851 |
|  | XP\_065052404\_2046-model1.5 | -18.3231 |
|  | XP\_065052404\_2046-model1.6 | -17.0257 |
| # cluster 2 - Size: 9 |  |  |
|  | XP\_065052404\_2046-model2 | -20.4832 |
|  | XP\_065052404\_2046-model2.1 | -19.8598 |
|  | XP\_065052404\_2046-model2.2 | -19.8408 |
|  | XP\_065052404\_2046-model2.3 | -18.54 |
|  | XP\_065052404\_2046-model2.4 | -18.0866 |
|  | XP\_065052404\_2046-model2.5 | -17.6904 |
|  | XP\_065052404\_2046-model2.6 | -16.4712 |
|  | XP\_065052404\_2046-model2.7 | -16.3464 |
|  | XP\_065052404\_2046-model2.8 | -15.5986 |
| # cluster 3 - Size: 7 |  |  |
|  | XP\_065052404\_2046-model3 | -19.9851 |
|  | XP\_065052404\_2046-model3.1 | -19.9401 |
|  | XP\_065052404\_2046-model3.2 | -18.7935 |
|  | XP\_065052404\_2046-model3.3 | -17.8125 |
|  | XP\_065052404\_2046-model3.4 | -17.5197 |
|  | XP\_065052404\_2046-model3.5 | -17.3719 |
|  | XP\_065052404\_2046-model3.6 | -13.0688 |
| # cluster 4 - Size: 4 |  |  |
|  | XP\_065052404\_2046-model4 | -19.3079 |
|  | XP\_065052404\_2046-model4.1 | -19.0865 |
|  | XP\_065052404\_2046-model4.2 | -17.8438 |
|  | XP\_065052404\_2046-model4.3 | -17.0462 |
| # cluster 5 - Size: 2 |  |  |
|  | XP\_065052404\_2046-model5 | -18.7964 |
|  | XP\_065052404\_2046-model5.1 | -13.8403 |
| # cluster 6 - Size: 1 |  |  |
|  | XP\_065052404\_2046-model6 | -18.3654 |
| # cluster 7 - Size: 1 |  |  |
|  | XP\_065052404\_2046-model7 | -17.8059 |
| # cluster 8 - Size: 4 |  |  |
|  | XP\_065052404\_2046-model8 | -17.0374 |
|  | XP\_065052404\_2046-model8.1 | -15.5345 |
|  | XP\_065052404\_2046-model8.2 | -13.8424 |
|  | XP\_065052404\_2046-model8.3 | -13.8399 |
| # cluster 9 - Size: 18 |  |  |
|  | XP\_065052404\_2046-model9 | -17.0358 |
|  | XP\_065052404\_2046-model9.1 | -16.6998 |
|  | XP\_065052404\_2046-model9.2 | -16.1578 |
|  | XP\_065052404\_2046-model9.3 | -16.1578 |
|  | XP\_065052404\_2046-model9.4 | -16.0562 |
|  | XP\_065052404\_2046-model9.5 | -16.0562 |
|  | XP\_065052404\_2046-model9.6 | -15.8567 |
|  | XP\_065052404\_2046-model9.7 | -15.5913 |
|  | XP\_065052404\_2046-model9.8 | -15.1597 |
|  | XP\_065052404\_2046-model9.9 | -14.7674 |
|  | XP\_065052404\_2046-model9.10 | -14.4411 |
|  | XP\_065052404\_2046-model9.11 | -14.052 |
|  | XP\_065052404\_2046-model9.12 | -14.0388 |
|  | XP\_065052404\_2046-model9.13 | -13.7717 |
|  | XP\_065052404\_2046-model9.14 | -13.044 |
|  | XP\_065052404\_2046-model9.15 | -13.0411 |
|  | XP\_065052404\_2046-model9.16 | -12.8112 |
|  | XP\_065052404\_2046-model9.17 | -12.0544 |
| # cluster 10 - Size: 2 |  |  |
|  | XP\_065052404\_2046-model10 | -16.9441 |
|  | XP\_065052404\_2046-model10.1 | -14.0083 |
| # cluster 11 - Size: 4 |  |  |
|  | XP\_065052404\_2046-model11 | -16.7461 |
|  | XP\_065052404\_2046-model11.1 | -16.2287 |
|  | XP\_065052404\_2046-model11.2 | -16.2287 |
|  | XP\_065052404\_2046-model11.3 | -14.3726 |
| # cluster 12 - Size: 3 |  |  |
|  | XP\_065052404\_2046-model12 | -16.4399 |
|  | XP\_065052404\_2046-model12.1 | -13.7404 |
|  | XP\_065052404\_2046-model12.2 | -11.88 |
| # cluster 13 - Size: 1 |  |  |
|  | XP\_065052404\_2046-model13 | -16.3559 |
| # cluster 14 - Size: 1 |  |  |
|  | XP\_065052404\_2046-model14 | -16.1311 |
| # cluster 15 - Size: 1 |  |  |
|  | XP\_065052404\_2046-model15 | -15.3201 |
| # cluster 16 - Size: 1 |  |  |
|  | XP\_065052404\_2046-model16 | -15.2353 |
| # cluster 17 - Size: 3 |  |  |
|  | XP\_065052404\_2046-model17 | -15.1393 |
|  | XP\_065052404\_2046-model17.1 | -14.7589 |
|  | XP\_065052404\_2046-model17.2 | -13.8022 |
| # cluster 18 - Size: 2 |  |  |
|  | XP\_065052404\_2046-model18 | -15.0124 |
|  | XP\_065052404\_2046-model18.1 | -12.2428 |
| # cluster 19 - Size: 1 |  |  |
|  | XP\_065052404\_2046-model19 | -14.6427 |
| # cluster 20 - Size: 1 |  |  |
|  | XP\_065052404\_2046-model20 | -14.5608 |
| # cluster 21 - Size: 10 |  |  |
|  | XP\_065052404\_2046-model21 | -13.4008 |
|  | XP\_065052404\_2046-model21.1 | -13.3578 |
|  | XP\_065052404\_2046-model21.2 | -13.2008 |
|  | XP\_065052404\_2046-model21.3 | -13.1127 |
|  | XP\_065052404\_2046-model21.4 | -12.3122 |
|  | XP\_065052404\_2046-model21.5 | -11.6447 |
|  | XP\_065052404\_2046-model21.6 | -11.5674 |
|  | XP\_065052404\_2046-model21.7 | -11.1722 |
|  | XP\_065052404\_2046-model21.8 | -10.8302 |
|  | XP\_065052404\_2046-model21.9 | -10.7455 |
| # cluster 22 - Size: 2 |  |  |
|  | XP\_065052404\_2046-model22 | -13.271 |
|  | XP\_065052404\_2046-model22.1 | -9.84187 |
| # cluster 23 - Size: 2 |  |  |
|  | XP\_065052404\_2046-model23 | -13.1187 |
|  | XP\_065052404\_2046-model23.1 | -13.0372 |
| # cluster 24 - Size: 1 |  |  |
|  | XP\_065052404\_2046-model24 | -12.6589 |
| # cluster 25 - Size: 1 |  |  |
|  | XP\_065052404\_2046-model25 | -12.4773 |
| # cluster 26 - Size: 2 |  |  |
|  | XP\_065052404\_2046-model26 | -12.3001 |
|  | XP\_065052404\_2046-model26.1 | -9.83043 |
| # cluster 27 - Size: 1 |  |  |
|  | XP\_065052404\_2046-model27 | -11.0638 |
| # cluster 28 - Size: 1 |  |  |
|  | XP\_065052404\_2046-model28 | -10.5476 |
| # cluster 29 - Size: 5 |  |  |
|  | XP\_065052404\_2046-model29 | -10.5158 |
|  | XP\_065052404\_2046-model29.1 | -10.3989 |
|  | XP\_065052404\_2046-model29.2 | -10.3313 |
|  | XP\_065052404\_2046-model29.3 | -10.2541 |
|  | XP\_065052404\_2046-model29.4 | -10.2154 |
| # cluster 30 - Size: 1 |  |  |
|  | XP\_065052404\_2046-model30 | -10.2279 |
| # cluster 31 - Size: 1 |  |  |
|  | XP\_065052404\_2046-model31 | -9.78227 |
|  |  |  |
